# Containment of Phytoplasma-Associated Plant Diseases by Antibiotics and Other Antimicrobial Molecules

**DOI:** 10.3390/antibiotics10111398

**Published:** 2021-11-14

**Authors:** Assunta Bertaccini

**Affiliations:** Department of Agricultural and Food Sciences, Alma Mater Studiorum—University of Bologna, 40127 Bologna, Italy; assunta.bertaccini@unibo.it

**Keywords:** phytoplasmas, plant disease, chemical control, induced resistance, molecular detection

## Abstract

Phytoplasmas are plant-pathogenic bacteria that infect many important crops and environmentally relevant plant species, causing serious economic and environmental losses worldwide. These bacteria, lacking a cell wall, are sensitive to antibiotics such as tetracyclines that affect protein synthesis mechanisms. Phytoplasma cultivation in axenic media has not been achieved for many strains; thus, the screening of antimicrobials must be performed using mainly *in vivo* materials. Some studies have investigated using *in vitro* phytoplasma-infected shoots, and several antimicrobials, including tetracyclines, have been tested. The screening of phytoplasma antimicrobials is important for the sustainable control of phytoplasma-associated diseases. The use of molecules with different modes of action such as ribosome inactivating proteins, plant hormones, and resistance inducers such as plasma-activated water, is advised, to avoid the use of antibiotics in agriculture and the possible emergence of resistant microbial strains.

## 1. Introduction

**Figure 1:**
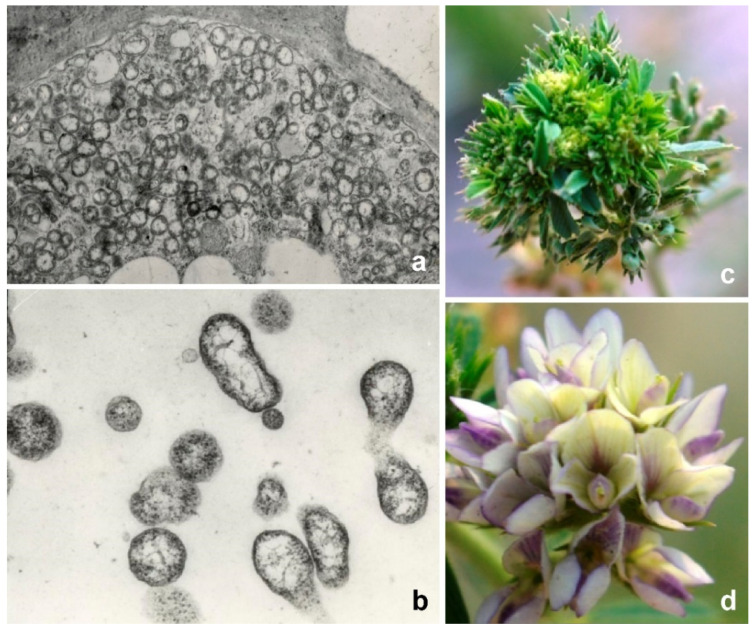
(**a**) transmission electron microscopy of phytoplasmas in sieve tissues (6000× magnification) (**b**); close up (10,000× magnification); (**c**) fenugreek (*Trigonella foenum-graecum*) inflorescence showing virescence and phyllody; (**d**) healthy inflorescence (courtesy J.N. Ahmad).

Phytoplasmas are mycoplasmas associated with several hundred plant diseases worldwide, including many diseases with important economic or environmental impacts. They are *Mollicutes, i.e*., prokaryotes lacking cell walls, found in plant phloem and insect hemolymph and are transmitted by insects, propagation materials, and seeds. The main symptoms associated with their presence are reduced plant growth, yellowing and decline, flower virescence, and malformations [1,2] (Figure 1). Historically, the use of antibiotics allowed the indirect confirmation of the phytoplasma’s role in several plant diseases [3] since they caused the disappearance, often temporary, of microbes and symptoms from the infected plants. The field control of phytoplasma diseases is mainly dependent on the use of insecticides against their insect vectors. However, this strategy is often ineffective because it cannot eliminate the source plants of these diseases [4]. Some studies have reported methods for the elimination of phytoplasmas from infected plants using shoot-tip culture, callus culture, heat therapy, and hot water treatment [5,6,7,8]; however, these methods cannot be used in the field. Only tetracycline antibiotics have demonstrated the ability to suppress phytoplasma symptoms and multiplication; however, it is currently difficult to achieve complete phytoplasma eradication from plants. Moreover, the antibiotic use is prohibited in many countries for agricultural use [9] and they may be hazardous to humans over time. For these reasons, non-antibiotic molecules were tested to assess whether they directly or indirectly reduce the phytoplasma presence and symptoms in infected plants. The use of antibiotic alternatives such as resistance inducers in open fields is difficult. *In vitro* systems could be an effective method to produce phytoplasma-free germplasm to be further multiplied in insect-proof conditions before its use in open fields. This strategy is reducing the environmental impact of insecticides/pesticide to manage the phytoplasma-associated diseases. In this review, approaches used for phytoplasma elimination in plants and *in vitro* shoots are summarized. The reduction of the presence of insect vectors in the field is almost ineffective as it is not feasible to eliminate all of them from the environment. Only the use of healthy plants can eliminate or reduce the spread of phytoplasma-associated diseases. 

## 2. Antibiotics

### 2.1. Field Application

Typical methods of antibiotic application in the field include foliar sprays, root immersion, soil drenching, and trunk injection. However, independently from the method used it was reported symptoms reoccurrence once the antibiotic treatments are suspended. In the urban areas of South Florida, antibiotics have been used in palms of high landscape value [10]. Nyland and Moller [11] first reported that tree decline and leaf curl as typical symptoms of pear decline could be prevented by injecting a solution of oxytetracycline hydrochloride into affected trees. Tetracycline was then successfully used in the United States of America to control pear decline [11]. However, this laborious, expensive and environmental impacting treatment has been discontinued because rootstocks less susceptible to the disease were used and pear cultivation was greatly reduced in the infected areas. Attempts to cure peach rosette and X-disease affected trees by chemotherapy in California using tetracycline injections into the trunk [12] verified initial phytotoxic effects together with a remission of symptoms in only a reduced number of treated plants. Antibiotics have been applied to diseased mulberry plants by foliar spray, root immersion of diseased seedlings, treatment of shoot cuttings before planting, treatment of budwoods before grafting, and treatment of stored and unsprouted shoots with some limited results [13]. In Europe, Seidl [14] reported that apple proliferation phytoplasma can be eliminated from budwood during summer if foliated apple bud sticks are exposed to oxytetracycline or chlortetracycline solution (100–200 ppm) for 24–48 h before using the buds for grafting. In India, tetracycline usage resulted in temporary remission of symptoms in brinjal infected with little leaf disease; however, this treatment could not eliminate the pathogen from the host plant [15,16]. Tetracycline root dip treatment of infected onion seedlings for 15 weeks at 7 days intervals resulted in phytoplasmas only being present in untreated infected plants [17]. *Physostegia virginiana* (false dragon head) was treated with a solution of oxytetracycline hydrochloride (100 ppm). Roots were dipped for 25 h in the dark, followed by washing and potting the plants under greenhouse conditions. The plants exhibited phytotoxicity only for the first three months. No phytoplasma symptoms appeared on the treated plants for two years, and the tissues tested negative to the presence of the bacterium under electron microscopy observations [18]. Ranunculus phytoplasma-infected plants showing strong stunting and rosette shape symptoms were watered with 300 mL oxytetracycline per plant three times per week for two months. It did not produce phytotoxicity at either concentration of 1 or 100 mg/L. A reduction in symptomatology was observed with the production of yellow flowers, typical of the healthy variety. Two weeks after the end of the treatment, the symptoms reappeared, confirming the bacteriostatic effect of the antibiotic [19]. Other molecules such as biophenicol, chloramphenicol, enteromycelin, lycercelin, paraxin, roscillin, camphicillin, oxytetracycline, chlorotetracycline, rose oil, clove oil, and eucalyptus oil were studied on brinjal cultivars infected with phytoplasmas. The application of the listed antibiotics did not show any significant effect in controlling brinjal little leaf disease. Moreover, no flowers and fruits were observed in any of the brinjal cultivars treated with antibiotics [20].

### 2.2. In Vitro Applications

Although treatment using oxytetracycline antibiotics suppresses phytoplasma propagation in infected plants cultured *in vitro*, high concentrations of antibiotics damage the tissues. Additionally, with this treatment, phytoplasma elimination is independent of the shoot tip size and overcomes the difficulties involved in excising very small meristems and their regeneration. Tetracyclines had a bacteriostatic effect on phytoplasmas in treated plants, but symptoms mostly reappeared after the transfer of plants to antibiotic-free medium [7,21]. Pear phytoplasma-infected explants were maintained for more than three years by micropropagation on Murashige and Skoog medium [22] supplemented with gibberellic acid, indole butyric acid, and benzyl amino purine. Phytoplasmas reached consistently higher concentrations in micropropagated explants than in samples from field-grown plants, although explants remained free from symptoms. Phytoplasmas were eliminated by incorporating 100 μg/mL oxytetracycline into the growth medium for a period of four weeks. These results have implications for plant propagation schemes of symptomless phytoplasma-infected explants obtained after micropropagation [23]. Severe phytotoxic effects on the growth and explant multiplication rate of potato *in vitro* culture were reported, as a function of increasing concentrations (up to 1024 mg/L) of chloramphenicol, streptomycin, and tetracycline in the culture medium [24]. In an attempt to eliminate almond witches’ broom from different almond varieties, no plant regeneration occurred from 1 cm microcuttings subjected to treatments with 50, 100, and 150 μg/mL oxytetracycline [25]. Phytotoxic effects of oxytetracycline were observed on grapevine, i.e., axillary buds were greatly affected when grown in a culture medium with 100 mg/L oxytetracycline [26]. Carvalho et al. [27] developed a methodology for eliminating the phytoplasma associated with frog skin disease in cassava using *in vitro* shoot culture combining thermotherapy and tetracycline treatments. Cuttings were exposed for a few minutes to different tetracycline concentrations, and then subjected to thermotherapy at temperatures ranging from 35 °C to 55 °C. Shoot tips of different sizes were excised (0.2, 0.4, 0.5, and 1.0 mm) and grown in a culture medium with 0, 5, 10, and 15 mg/L tetracycline for 60 days. PCR analysis showed that the phytoplasma was eliminated in 100% of the previously infected plants seven months after being transferred in the field. Micropropagated periwinkle shoots infected with ‘*Candidatus* Phytoplasma rubi’ (strain RuS) were maintained for 90 days in solid culture medium [28]; 0.5 mL distilled water containing tetracycline hydrochloride (MW 480.9) diluted 1:100 or 9 CH homeopathic dynamizations of tetracycline were added once per week. In all the treatments, the infected shoots showed a high mortality rate, and those surviving were still phytoplasma positive after testing with nested PCR with phytoplasma 16SrV group-specific primers. The shoots grown on 9 CH dynamized tetracycline showed no symptoms while those grown on the media containing diluted tetracycline or sterile distilled water did, suggesting some enhancement of the plant defenses in those shoots [29]. A comprehensive screening method was developed that uses a plant-phytoplasma co-culture system to evaluate antibiotics, using lower concentrations (100–120 ppm), to reduce the damage to plant tissues and sustain the defense response of plant cells to phytoplasmas [30]. Using this system, more than 40 antibiotics were tested comprising several classes such as peptide, sulfonamide, quinolone, rifamycin, tetracycline, phenicol, and macrolide. A number of these molecules were shown to decrease the concentration of the onion yellows wild phytoplasma strain in the micropropagated shoots. Moreover, phytoplasmas were eliminated from infected shoots after four-month treatment by the application of both tetracycline and rifampicin targeting phytoplasma protein and RNA, respectively.

**Figure 2:**
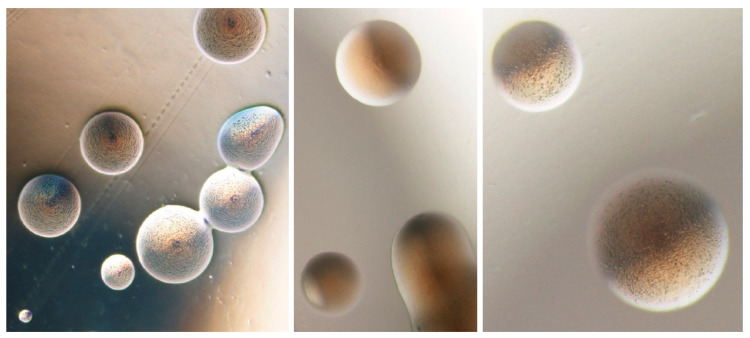
Phytoplasma colonies in solid medium CB [31] taken using optical microscope at 40× magnification (courtesy N. Contaldo).

A preliminary evaluation of the *in vitro* antimicrobial activity in phytoplasma colonies (Figure 2) was performed using seven antibiotics against two phytoplasma isolates from coconut plants infected by lethal yellowing disease [31,32]. The standard disc diffusion method was employed using a selection of the antibiotics previously demonstrated able to reduce the phytoplasma presence in micropropatated shoots [30]. Rifampicin, 5-fluorouracil, tetracycline, tobramycin, polymyxin B, and cephalexin hydrate inoculated with 10^8^ CFU/mL of phytoplasma isolates were used. The results of the antibiotic susceptibility tests revealed that tobramycin exhibited the maximum of activity against the tested phytoplasma isolates, followed by polymyxin B and tetracycline. The isolates displayed intermediate susceptibility to 5-fluorouracil but were completely resistant to cephalexin hydrate and rifampicin.

## 3. Antimicrobial Molecules

Experiments performed employing diverse kind of molecules as alternative to an-tibiotics such as kinetins showed that they were ineffective in phytoplasma elimination [33,34]. Also, a β-aminobutyric acid treatment of phytoplasma-infected periwinkle shoots [35] proved to be ineffective, while putrescine, spermidine or spermine caused various alterations in the phytoplasma ultrastructure, which could account for reduced multiplication and movement of the pathogens in the infected plants [35]. A slower development of symptoms was also observed in polyamine treated shoots compared to infected controls [36]. The effect of exogenously supplemented auxins was investigated on greenhouse-grown and shoot-tip cultures of periwinkle infected with *Spiroplasma citri* and phytoplasmas [37] and in *in vitro*-grown ‘*Ca.* P. trifolii’-related strain infected plants of the same species [38]. An increase in the endogenous concentration of indole-3-acetic acid (IAA) in phytoplasma-infected plants and a reduced number of phytoplasmas in ultrathin sections of infected plant cells was observed after treatment with high hormone concentrations. When *in vitro* shoots of periwinkle infected with different strains of ‘*Ca*. Phytoplasma’ such as ‘*Ca*. P. pruni’ (strain KVI, clover phyllody) and ‘*Ca*. P. asteris’ (strain HYDB, hydrangea phyllody) were exposed to IAA or indole-3-butyric acid, both auxins induced recovery of the symptoms in phytoplasma infected shoots. The latter was more effective [34]. It was observed that recovery was dependent on the ‘*Ca*. Phytoplasma’ strain, the duration of the treatment and the concentration and type of auxin. On the contrary, ‘*Ca*. P. ulmi’ (strain EY-C) and ‘*Ca*. P. solani’ (strain SA-1) persisted in the host tissues despite the shoots appeared to be symptomless [39]. The susceptibility of ‘*Ca*. P. mali’ to several chemical or synthetic antimicrobial agents as nisin, esculetin, pyrithione and chloramphenicol as molecules having different target activities was also evaluated. The activity of these molecules was compared with the one of two antibiotics (tetracycline and enrofloxacin) in *in vitro* grown infected apple shoots by adding them to the medium at 100, 500, 1000 ppm; nisin and pyrithione which were tested at 10, 100 and 500 ppm. The qPCR results showed that the phytoplasma was not detectable after one and two months only in presence of pyrithione at 10 and 100 ppm. Moreover, some other products reduced the concentration of phytoplasmas after two months. Shoots died or withered on media enriched with essential oils; especially when they were used at concentration of 500 and 1000 ppm [40]. 

Experiments carried out with PAPII [41], a ribosome-inactivating protein (RIP) extracted from *Phytolacca americana,* showed some efficacy in phytoplasma elimination in micropropagated infected plant shoots. RIPs are specific N-ß-glycosidases isolated from plants and share the ability to hydrolyze the N-glycosidic bond of a single adenosine present in a conserved sequence of the major RNA of ribosomes. Micropropagated periwinkle shoots infected with a ‘*Ca.* P. asteris’ strain (hydrangea virescence, HyV strain) were employed. Preliminary tests carried out on shoots immersed in sterile water containing decreasing concentrations of PAPII showed an increased number of necrotic shoots after the first 48 h of exposure. However, when PAP-II was added to the medium, no phytotoxicity was detectable, regardless of the exposure time. Batches of 1–3 cm long shoots were treated with serial dilutions of PAPII for 15 to 150 days. Only the 4% of infected shoots grown on a medium enriched with PAPII at dilutions of 1:10 and 1:100 for 15–150 days were found to be free from phytoplasmas. The elimination rate appeared to be related to both PAPII concentration and exposure time. The percentage of phytoplasma-free shoots ranged from 40 to 50% in dilutions ranging from 1:10 to 1:1000 and for periods between 50 and 150 days. The optimum treatment to achieve phytoplasma elimination was using 1:1000 dilutions for approximately 3 months [42].

**Figure 3:**
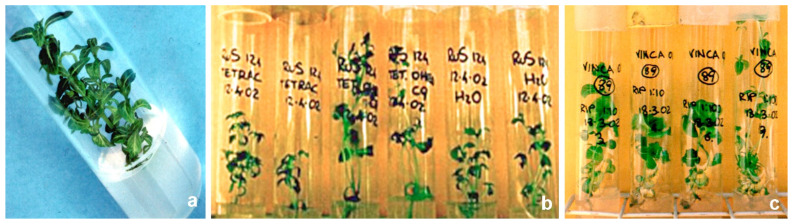
Periwinkle shoots in micropropagation: (**a**) untreated shoot; (**b**) phytoplasma-infected shoots treated with tetracyclines (the two central shoots were treated with 9 CH dynamized tetracycline); (**c**) shoots treated with PAPII.

Further trials to eliminate phytoplasmas from infected shoots were carried out using PAPII on micropropagated periwinkle shoots infected with ‘*Ca*. P. asteris’ (FE1 and O-1), ‘*Ca*. P. pruni’ (RA), and ‘*Ca*. P. rubi’ (Rus) strains maintained in collection [43,44]. PAPII was used at three dilutions (1:10, 1:100, and 1:1000) and the treated shoots were compared to infected shoots of the same strains grown in clean medium [33]. The shoots were maintained in a growth chamber at 24°C with 16 h light for 90 days and tested by nested PCR to verify phytoplasma presence. After this period the great majority of the survived shoots was still positive for phytoplasma presence and shoots were therefore subject to a second 90-day treatment under the same conditions as before. The highest growing shoot percentage was observed for the ‘*Ca*. P. rubi’ strain grown on a 1:10 dilution of PAPII. The phytoplasma elimination rate increased after the second treatment and at the higher PAPII concentration. No phytoplasma elimination was observed in the shoots grown in the 1:1000 dilution in either the short- or longterm treatments. A further experiment was performed on two other strains of ‘*Ca*. P. asteris’ (HyV and O-1) for a total of 90 days using the same tetracycline dilutions. Only a small number of shoots resulted negative for phytoplasma presence (Figure 3) [29].

## 4. Plant Resistance Inducers

**Figure 4:**
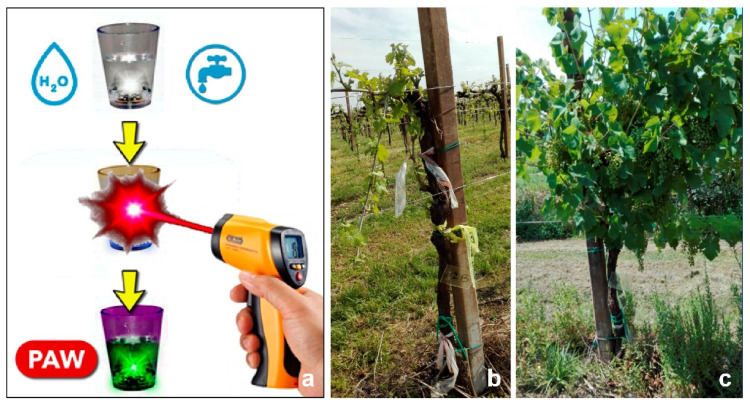
(**a**) scheme to produce PAW; (**b**) grapevine plant before and (**c**) after the PAW application for a period of three years.

A technology based on plasma-activated water (PAW), characterized by the presence of reactive oxygen and nitrogen species (RONS) in liquid, was tested on phytoplasma-infected micropropagated shoots and plants, in orchards and in greenhouse cultivation systems, to evaluate its effectiveness as a resistance inducer [45]. The exposure of sterile distilled water (SDW) to a cold atmospheric pressure plasma (CAP) caused a reduction in pH and the production of RONS that induced plant defense responses. To evaluate the effectiveness of PAW to control the phytoplasma-associated disease grapevine yellows, infected plants were treated in open-field and greenhouse conditions. The qualitative and quantitative yield parameters, the phytoplasma presence, and the gene expression were evaluated. The results showed that PAW enhanced the plant defense mechanisms and, as demonstrated in the field trials, improved the health status of the treated plants [46]. In a preliminary field trial performed on 120 plants from 17 vineyards, with treatments performed in April, June, and July for three years, treated plants showed a slight reduction and a delay in the phytoplasma symptom appearance, which allowed the plants to carry their productive load (Figure 4).

The analyses also verified that PAW treatment reduced the number of infected plants [47,48]. The quantitative yield parameters, measured after one year of treatment on 50 infected plants, confirmed a plant fitness improvement in terms of the number of grapevine clusters per plant and 100-berry weights. PAW-treated plants registered a significantly higher average number of clusters than controls, with an increased average berry weight [46]. These findings were unexpected due to the lack of positive correlation in resistance induction and plant fitness reported for other plant elicitors. It is known that, in plants, the secondary metabolite activation under stress has an energetic cost and leads to a reduction in growth and production. Considering the multiple variables present in the field, it is not yet possible to state the practical impact of this containment tool, but it may certainly be enclosed in a new and eco-sustainable management strategy for phytoplasma-associated diseases. Transcriptional and post-transcriptional molecular analyses highlighted PAW’s ability to enhance the expression of genes encoding the main enzymes involved in the phytoalexin biosynthetic pathway (alkaloids and stilbenes) in grapevine and periwinkle, and to modulate some of the stress response genes through the regulation of miRNAs [49].

## 5. Discussion and Conclusions

The elimination of phytoplasmas by *in vitro* shoot culture using anti-microbial agents such as tetracycline and rifampicin enabled the production of phytoplasma-free nursery stocks, contributes to the preservation of mother plants and biodiversity, and supports the international distribution of botanical resources. The most promising approach to plant sanitation from phytoplasma infection is the combination of *in vitro* (chemo)therapy followed by the micropropagation of phytoplasma-free shoots. This method allows the production of plants to stock clean plantations. In this approach, the use of sensitive and reliable testing methods [50,51,52] for verifying the phytoplasma elimination from the plant material after the treatment is key for a successful production of these plants. The application of plant resistance inducers such as PAW, supports the production of asymptomatic shoots and plants, where a mutualistic host–pathogen interaction takes place with the production of healthy-looking plants. This situation could also be permanent, especially without further stress to the plants; however, due to the presence of insect vectors in the environment and propagation techniques such as the grafting, the phytoplasmas can still be transferred to other host plants or other plant species. In the latter cases, bacteria presence can lead to dangerous, unwanted and unpredictable epidemics. The screening for anti-phytoplasma molecules or tools is therefore a key step towards sustainable field control and management of phytoplasma-associated diseases. The use of molecules with different modes of action such as plant hormones, ribosome inactivating proteins, plant resistance inducers (PAW) is advisable to avoid the use of antibiotics in agriculture and therefore the possible emergence of resistant microbial strains [9]. The comparison of proteins targeted by antimicrobials and conserved among various phytoplasma strains, may provide an indication of the potential effectiveness of new or already used molecules against a range of different phytoplasmas.
